# Evaluation of *Emblica officinalis* fruit powder as a growth promoter in commercial broiler chickens

**DOI:** 10.14202/vetworld.2016.207-210

**Published:** 2016-02-27

**Authors:** A. P. Patel, S. R. Bhagwat, M. M. Pawar, K. B. Prajapati, H. D. Chauhan, R. B. Makwana

**Affiliations:** 1 Department of Animal Nutrition, College of Veterinary Science and Animal Husbandry, Sardarkrushinagar Dantiwada Agricultural University, Banaskantha, Gujarat, India; 2 Livestock Research Station, Sardarkrushinagar Dantiwada Agricultural University, Banaskantha, Gujarat, India; 3 Department of Livestock Production and Management, College of Veterinary Science and Animal Husbandry, Sardarkrushinagar Dantiwada Agricultural University, Banaskantha, Gujarat, India

**Keywords:** broiler chickens, *Emblica officinalis*, feed conversion ratio, growth performance

## Abstract

**Aim:**

The present study was conducted to evaluate the dietary addition of *Emblica officinalis* (Amla) fruit powder as a growth promoter in commercial broiler chickens.

**Materials and Methods:**

An experiment was conducted on 135 commercial broiler chicks (Ven-Cobb 400 strain) divided into three groups with three replicates of 15 chicks each. Three treatment groups were as follows – T_1_: Basal diet as per BIS standards; T_2_: Basal diet supplemented with 0.4% of *E. officinalis* fruit powder; and T_3_: Basal diet supplemented with 0.8% of *E. officinalis* fruit powder.

**Results:**

The average body weights at the end of the 6^th^ week were significantly higher (p<0.05) in groups T_2_ and T_3_ compared to group T_1_. Feed intake, feed conversion ratio and feed cost per kg live weight production were similar among the treatment groups. The net profit per bird was the highest in group T_2_ (Rs. 19.22/bird) followed by group T_3_ (Rs. 17.86/bird) and the lowest in group T_1_ (Rs. 14.61/bird).

**Conclusion:**

Based on the results of the present study, it was concluded that dietary addition of *E. officinalis* (Amla) fruit powder had a positive effect on growth performance and net profit per bird in commercial broiler chickens.

## Introduction

Growth promoters are chemical and biological substances which are added to diet with the aim to improve the growth, utilization of nutrients and in this way realize better production and financial results. Their positive effect can be expressed through better appetite, improved body weight and feed conversion ratio (FCR), stimulation of the immune system and increased vitality, regulation of the intestinal microflora, etc. In any case, expected results of the use of these additives are increased financial returns over the cost of production. Furthermore, due to ban on the use of antibiotic growth promoters in poultry, herbal preparations have been tried as feed additives as an alternative to antibiotics to increase feed efficiency and growth rate in broiler chickens [1].

In the last decade, herbal feed additives have attracted the attention of scientists as useful resource for improving productivity. Besides, these herbs are natural component and do not have any side effects like residues in meat products. Amla (*Emblica officinalis*) fruit powder is one of the herbs which have potential to boost broiler production. Amla is extensively cultivated all over India. The fruits of the plants are used in Ayurveda as a potant rasayana (revitalisers, biological response modifiers) in which the amla was added as anti-stress agent. Phyto-chemical analysis of amla fruit powder provided evidence of presence of the medicinally important bioactive compounds which can be exploited beneficially to improve productivity in broilers.

*Emblica officinalis* (Amla) is one of the richest sources of ascorbic acid, minerals, amino acids, tannins, and phenolic compounds [2]. Rapid growth rate in commercial broilers accelerate the metabolic rate and make them vulnerable to oxidative stress owing to increased free radical generation [3]. Gallic acid and tannic acids are the phenolic acids present in *E. officinalis* contribute to the antioxidant activity, in addition, to ascorbic acid [4]. Therefore, the present study was conducted to evaluate the dietary addition of *E. officinalis* (Amla) fruit powder as a growth promoter in commercial broiler chickens.

## Materials and Methods

### Ethical approval

This research was carried out after approval of Institutional Animal Ethics Committee of College of Veterinary Science and Animal Husbandry, Sardarkrushinagar Dantiwada Agricultural University, Gujarat.

### Experimental design

A total of 1351-day-old unsexed broiler chicks (Ven-Cobb 400 strain) purchased from a local hatchery were weighed and randomly assigned to one of three treatments with three replicates of 15 chicks based on a completely randomized design. Three treatments were as follows – T_1_: Basal diet as per BIS standards; T_2_: Basal diet supplemented with 0.4% of *E. officinalis* fruit powder; and T_3_: Basal diet supplemented with 0.8% of *E. officinalis* fruit powder.

### Feeding and management procedures

Broilers were raised on deep litter housing system for 6 weeks. Feed and water were provided *ad libitum* throughout the experiment. Broilers were fed in three phases, *viz*., pre-starter (0-10 days), starter (11-21 days), and finisher (22-42 days) ration as per BIS [5] specifications. Chicks were individually weighed at weekly intervals. Feed consumption and FCR (FCR=feed intake/weight gain) were calculated at weekly intervals. Mortality was recorded daily. The feed cost-economics of broiler production in different treatment groups was calculated based on the current market price of various particulars.

### Chemical and statistical analysis

The ingredients and chemical composition of basal diet used during experimental feeding are given in Table-1. Samples of feeds were milled to pass through a 1mm sieve and then analyzed for the chemical composition according to standard procedures of the AOAC [6] methods. The experimental data were statistically analyzed using SPSS software (version 16.0, SPSS Inc., Chicago, USA) as per procedures of Snedecor and Cocharan [7]. The significant differences among the tested means were tested with Duncan's multiple range test [8], and significance was declared at p<0.05.

**Table-1:** Ingredients and chemical composition of basal diet used during experimental feeding.

Attributes	Pre-starter	Starter	Finisher
Ingredients (%)			
Yellow maize	51.28	52.05	57.00
Soybean meal	40.21	38.43	33.03
Vegetable oil	4.56	5.6	6.38
Dicalcium phosphate	1.93	1.97	1.71
Common salt	0.35	0.35	0.35
Limestone	0.97	1.01	0.93
Maduramycine	0.05	0.05	0.05
Lipocare^1^	0.10	0.10	0.10
L-lysine	0.17	0.15	0.14
DL-methionine	0.15	0.15	0.07
Vitamin premix^2^	0.05	0.05	0.05
Mineral premix^3^	0.20	0.20	0.20
Total	100.02	100.01	100.01
Nutrient composition (%)			
Dry matter	94.63	94.71	94.32
Crude protein	23.21	21.64	19.62
Ether extract	2.75	3.46	4.20
Crude fiber	4.98	5.03	5.01
Total ash	5.72	6.63	5.07
ME (kcal/kg)	2998.2	3075.1	3185.6

1 Lecithin treated with co-enzyme

2 Provides per kg of diet: 12,500 IU vitamin A; 2500 IU vitamin D_3_; 12 mg vitamin E; 1.5 mg vitamin K; 1.5 mg vitamin B_1_; 5 mg; vitamin B_2_; 2 mg vitamin B_6_, 15 mcg vitamin B_12_; 15 mg niacin, 10 mg pantothenic acid and 0.5 mg folic acid

3 Provides per kg of diet: 50 mg iron; 10 mg copper; 80 mg zinc; 80 mg manganese; 1 mg iodine and 0.2 mg selenium. ME: Metabolizable energy

## Results and Discussion

### Growth performance

The data on growth performance is presented in Table-2. The average body weights of the birds at the end of the 6^th^ week were higher (p<0.05) in groups T_2_ (2186.00±19.48 g) and T_3_ (2170.60±14.62 g) compared to group T_1_ (2076.30±22.27 g). In the present study, the birds supplemented with *E. officinalis* fruit powder at the rate of 0.4% and 0.8% had higher (p<0.05) overall body weights and weekly body weight gain at the end of 6^th^ week compared to un-supplemented group. The higher body weights observed in *E. officinalis* supplemented groups may be attributed to anabolic and antioxidant effect of ascorbic acid, gallic acid and tannic acids present in *E. officinalis* [9]. Similar findings were reported by Maini *et al*. [10], Patil *et al*. [11], Kumari *et al*. [12] Patil *et al*. [13]. In another studies, Sujatha *et al*. [14] and Kumar *et al*. [15] reported increase in body weight when birds were supplemented with polyherbal feed premix containing *E. officinalis*.

**Table-2:** Average weekly body weights (g/bird) and body weight gain (g) of broiler chicks under different treatment groups.

Weeks	Treatments			Significance

	T_1_	T_2_	T_3_	
Average weekly body weights (g/bird)				
Day old	47.04±0.56	46.22±0.36	46.71±0.47	NS
I	129.49±2.48	131.93±2.35	130.47±1.59	NS
II	394.36^a^±5.53	425.38^b^±6.62	423.34^b^±3.48	0.012*
III	699.73^a^±8.48	776.76^b^±13.97	750.32^b^±6.07	0.016*
IV	1101.50^a^±14.58	1176.80^b^±17.15	1152.60^b^±10.99	0.032*
V	1583.20^a^±20.43	1653.90^b^±25.13	1642.20^b^±11.74	0.027*
VI	2076.30^a^±22.27	2186.00^b^±19.48	2170.60^b^±14.62	0.003**
Average weekly weight gain (g)				
I	82.4±2.41	85.7±2.33	83.8±1.56	NS
II	265.5^a^±5.92	293.4^b^±6.79	293.1^b^±3.69	0.009**
III	305.4^a^±8.13	351.4^b^±14.72	327.0^ab^±5.98	0.026*
IV	403.2±12.96	400.0±19.01	402.3±11.49	NS
V	481.7±22.33	470.7±24.48	489.6±13.27	NS
VI	493.1±25.45	532.1±25.55	528.4±11.07	NS
0-VI	2029.3^a^±22.33	2139.7^b^±19.43	2123.8^b^±14.56	0.002**

a,b,c Means bearing different superscripts in a row differ significantly

* p<0.05

** p<0.01

### Feed intake and FCR

Feed intake and FCR were non-significant (p>0.05) among the treatment groups (Table-3). Dietary supplementation of *E. officinalis* at both levels (0.4% and 0.8%) did not have any adverse (p=0.307) effect on feed intake in broilers.Our findings are in agreement with prior studies [15,16] which demonstrated that supplementation of *E. officinalis* had no effect of feed intake and FCR. In contrastPatil *et al*. [13] reported that significant increase in feed intake when birds were supplemented with either *E. officinalis* fruit powder alone or in form of poly-herb.

**Table-3:** Average weekly feed intake (g/bird) and FCR in broilers under different treatment groups.

Weeks	Treatments			Significance

	T_1_	T_2_	T_3_	
Average weekly feed intake (g/bird)				
I	113.9±3.12	116.1±1.12	114.0±0.45	NS
II	396.5±9.23	419.6±11.58	416.4±4.07	NS
III	501.6^a^±9.45	536.5^b^±1.18	510.0^a^±4.53	0.016*
IV	693.3±3.10	693.0±6.10	697.3±5.61	NS
V	898.5±19.30	855.2±42.34	880.8±18.74	NS
VI	1018.2^a^±15.60	1087.9^b^±7.77	1071.0^b^±11.83	0.018*
0-VI	3622.1±38.54	3708.4±45.34	3689.4±26.89	NS
Average weekly FCR				
I	1.38±0.03	1.36±0.05	1.36±0.04	NS
II	1.49±0.04	1.43±0.01	1.42±0.01	NS
III	1.64^b^±0.01	1.53^a^±0.04	1.56^a^±0.01	0.034*
IV	1.72±0.01	1.73±0.02	1.73±0.02	NS
V	1.86±0.02	1.82±0.03	1.80±0.01	NS
VI	2.06±0.03	2.05±0.01	2.03±0.01	NS
0-VI	1.80±0.02	1.75±0.02	1.74±0.01	NS

a,b,c Means bearing different superscripts in a row differ significantly (p<0.05). FCR=Feed conversion ratio

### Return over feedcost

The cost of feed per kilogram of live weight production was similar among the treatment groups(Table-4). The profit per bird was the highest (p<0.05) in group T_2_ (Rs. 19.22/bird) followed by group T_3_ (Rs. 17.86/bird) and lowest in group T_1_ (Rs. 14.61/bird). The higher net profit per bird in *E. officinalis* supplemented groups attributed to higher body weights compared to the un-supplemented group and similar feed intakes among all the treatment groups.

**Table-4:** Economics of broiler production and mortality in different treatment groups.

Particulars	Treatments		

	T_1_	T_2_	T_3_
Feed cost/kg live weight production (Rs.)	45.36	44.51	45.00
Net profit/bird (Rs.)	14.61	19.22	17.86
Mortality (%)	4.44	2.22	2.22

### Mortality

The mortality was 4.4%, 2.2% and 2.2% in T_1_, T_2_ and T_3_ groups, respectively (Table-3). The data indicated that the percent mortality is well within the normal limit, i.e., below 5%. However, the percent mortality in *E. officinalis* fruit powder supplemented groups (T_2_ and T_3_) were lower indicating better livability of birds as compared to T_1_ group, which may be due to immune-modulatory property of bioactive compounds present in *E. officinalis*. Similarly, Kumar and Singh [17] reported that mortality was reduced in birds supplemented with either *E. officinalis* fruit powder or its mixture with other herbs.

## Conclusion

Results indicated that dietary addition of *E. officinalis* (Amla) fruit powder at the rate of 0.4% and 0.8% had higher growth rate and net profit per bird in commercial broiler chickens. Though *E. officinalis* supplementation had shown positive response in the present study, but it needs to be tested at different supplemental levels and in different ration compositions to get the best results.

## Authors’ Contributions

SRB and MMP designed and supervised the experiment. APP carried out the experimental work. RBM carried out laboratory analysis of feed samples. MMP, KBP and HDC did the data analysis and drafted the manuscript. All authors read and approved the final manuscript.
